# A genome-wide CRISPR/Cas9 screen reveals that the aryl hydrocarbon receptor stimulates sphingolipid levels

**DOI:** 10.1074/jbc.AC119.011170

**Published:** 2020-02-06

**Authors:** Saurav Majumder, Mari Kono, Y. Terry Lee, Colleen Byrnes, Cuiling Li, Galina Tuymetova, Richard L. Proia

**Affiliations:** Genetics of Development and Disease Branch, NIDDK, National Institutes of Health, Bethesda, Maryland 20892

**Keywords:** sphingolipid, aryl hydrocarbon receptor (AhR) (AHR), myelin, transcription factor, ceramide, axon myelination, CRISPR/Cas9, serine palmitoyltransferase small subunit A (SPTSSA), gene regulation, membrane lipid

## Abstract

Sphingolipid biosynthesis generates lipids for membranes and signaling that are crucial for many developmental and physiological processes. In some cases, large amounts of specific sphingolipids must be synthesized for specialized physiological functions, such as during axon myelination. How sphingolipid synthesis is regulated to fulfill these physiological requirements is not known. To identify genes that positively regulate membrane sphingolipid levels, here we employed a genome-wide CRISPR/Cas9 loss-of-function screen in HeLa cells using selection for resistance to Shiga toxin, which uses a plasma membrane-associated glycosphingolipid, globotriaosylceramide (Gb3), for its uptake. The screen identified several genes in the sphingolipid biosynthetic pathway that are required for Gb3 synthesis, and it also identified the aryl hydrocarbon receptor (AHR), a ligand-activated transcription factor widely involved in development and physiology, as being required for Gb3 biosynthesis. AHR bound and activated the gene promoter of serine palmitoyltransferase small subunit A (*SPTSSA*), which encodes a subunit of the serine palmitoyltransferase that catalyzes the first and rate-limiting step in *de novo* sphingolipid biosynthesis. *AHR* knockout HeLa cells exhibited significantly reduced levels of cell-surface Gb3, and both *AHR* knockout HeLa cells and tissues from *Ahr* knockout mice displayed decreased sphingolipid content as well as significantly reduced expression of several key genes in the sphingolipid biosynthetic pathway. The sciatic nerve of *Ahr* knockout mice exhibited both reduced ceramide content and reduced myelin thickness. These results indicate that AHR up-regulates sphingolipid levels and is important for full axon myelination, which requires elevated levels of membrane sphingolipids.

## Introduction

Sphingolipids (SLs)^2^ are an essential class of lipids involved in diverse biological functions. They function as integral components of eukaryotic membranes, and their metabolic intermediates, such as sphingosine-1-phosphate (S1P), are bioactive signaling molecules (1). SLs are critical for embryonic growth, development, and the maintenance of normal physiology (2–6).

Unlike the case with other major classes of lipids, such as sterols and glycerolipids, our understanding of how SL synthesis is regulated is limited. Homeostatic control of the *de novo* pathway is now known to be achieved through feedback regulation by the ORMDL family of proteins (7, 8). These small endoplasmic reticulum proteins sense ceramide levels and inhibit serine palmitoyltransferase (SPT), which catalyzes the first committed step in *de novo* SL biosynthesis (7). A second homeostatic control mechanism involves ceramide sensing via sphingomyelin synthase–related enzyme (9). However, certain developmental processes, such as myelination of axons and formation of the skin permeability barrier, require high levels of specific SLs (10–13). The underlying mechanisms that mediate the increased synthesis of SLs needed to fulfill such specialized roles are not understood.

In our investigation to determine genes that positively regulate membrane SL levels, a genome-wide CRISPR/Cas9 screen identified the aryl hydrocarbon receptor (AHR), a transcription factor involved in diverse developmental and physiological processes (14–19). We showed that AHR is required for normal mRNA expression of several key SL biosynthetic genes, as well as regulating SL levels in cells and tissues. In addition, we found that AHR is needed for full myelination of sciatic nerve, a developmental process that depends on high levels of membrane SLs (6).

## Results

### Genome-wide CRISPR/Cas9 screen for positive regulators of SL biosynthesis

**Figure 1.**
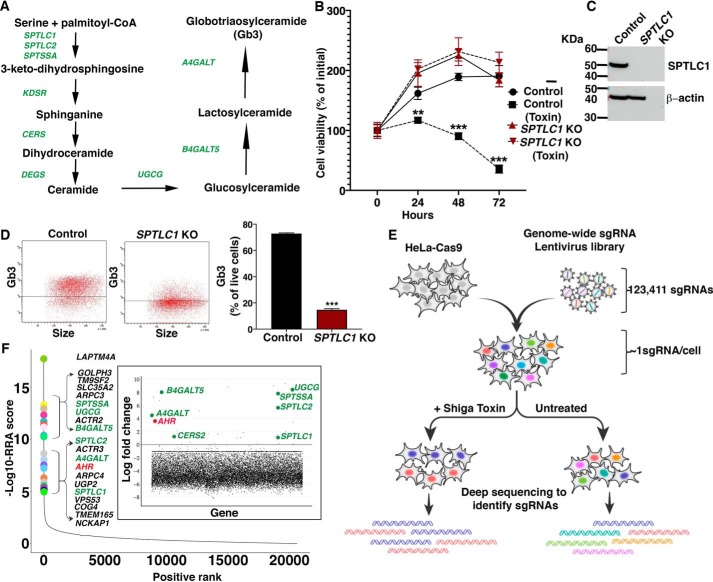
**Genome-wide CRISPR/Cas9 screen for positive regulators of SL biosynthesis.** *A*, *schematic* showing SL biosynthetic pathway leading to the production of Gb3. Genes required for the pathway are shown in *green. B*, cell viabilities of WT HeLa cells (control) and *SPTLC1* KO HeLa cells with or without Shiga toxin (2 ng/ml) treatment for 72 h. Data are expressed as mean ± S.D. (*error bars*) (*n* = 3). *C*, representative immunoblot analysis of SPTLC1 protein expression in WT (control) and *SPTLC1* KO HeLa cells. β-Actin was used as a loading control. *D*, flow cytometry analysis of WT (control) and *SPTLC1* KO HeLa cells stained with anti-CD77 antibody. *Left*, representative dot plot of cell-surface expression of Gb3. *Right*, quantification of Gb3 expression (mean ± S.D.; *n* = 3). Unpaired *t* test was used: **, *p* ≤ 0.01; ***, *p* ≤ 0.001. *E*, *schematic representation* of the genome-wide CRISPR/Cas9 screening strategy undertaken to identify genes required for Shiga toxin resistance in HeLa cells. *F*, *scatterplot* showing the ranking of positively selected genes from MAGeCK analysis. The *x* axis indicates positive ranking of individual genes, and the *y* axis indicates −log_10_ values of corresponding robust ranking aggregation (*RRA*) score. The 20 top-ranking genes are *highlighted* and *labeled. Inset*, *scatterplot* showing log -fold change for all of the genes. Genes involved in the SL biosynthetic pathway that show positive log -fold change in the toxin-resistant group are *highlighted* in *green. AHR* is *highlighted* in *red*.

Shiga toxin binds specifically to the cell-surface trisaccharide glycosphingolipid receptor, globotriaosylceramide (Gb3), and causes cell death after uptake as a result of toxin-mediated protein synthesis inhibition (20–22). Production of the Gb3 receptor requires the *de novo* ceramide synthesis pathway, as well as three glycosyltransferases (UGCG, B4GALT5, and A4GALT) for the addition of the trisaccharide structure to ceramide (Fig. 1*A*). Because the level of cell-surface Gb3 on HeLa cells correlates with their sensitivity to the toxin (23, 24), we reasoned that selection of Shiga toxin-resistant HeLa cells after transduction of a genome-scale knockout (KO) library could yield not only the biosynthetic genes required for Gb3 synthesis, as has been previously shown (25–27), but also identify novel positive regulatory genes that control membrane SL levels.

To undertake the screen, we used HeLa cells stably expressing Cas9. Shiga toxin at 2 ng/ml substantially reduced viability of these HeLa cells over a 72-h period (Fig. 1*B*). To validate that the Shiga toxin–dependent reduction in viability depended on the SL *de novo* synthesis pathway, we generated *SPTLC1* KO HeLa cells, which were deficient in an essential subunit of the SPT enzyme complex (4) (Fig. 1*C*). Compared with control HeLa cells, *SPTLC1* KO HeLa cells were resistant to Shiga toxin treatment (Fig. 1*B*) and expressed significantly decreased cell-surface Gb3 (Fig. 1*D*), indicating that Shiga toxin resistance was due to the loss of Gb3 receptor caused by the deletion of *SPTLC1* (Fig. 1*A*).

Based on these results, we established a genome-wide loss-of-function screen using Shiga toxin selection to identify genes that positively regulate membrane SL levels. We used a lentivirus genome-scale CRISPR/Cas9 knockout (GeCKO) library with sgRNAs targeting 19,050 genes (28). HeLa cells stably expressing Cas9 transduced with the library at a multiplicity of infection (MOI) of ∼0.25 were grown in the presence or absence of Shiga toxin, and genomic DNA of toxin-treated and untreated cell pools was subjected to deep sequencing to identify the abundance of sgRNAs (Fig. 1*E*).

Relative abundance of sgRNAs present in toxin-treated over untreated cells were compared using the MAGeCK algorithm (29). Genes represented by individual sgRNAs were ranked using the modified “robust ranking aggregation” score from MAGeCK analysis (Fig. 1*F* and Table S1). Multiple genes involved in the Gb3 biosynthetic pathway were identified. The 20 top-ranking genes included *SPTSSA*, *UGCG*, *B4GALT5*, *SPTLC2*, *A4GALT*, and *SPTLC1*. These biosynthetic genes, along with *CERS2,* also showed relatively high positive log -fold changes in the toxin-treated cells compared with untreated cells (Fig. 1*F*, *inset*), which is consistent with previous reports (25, 26) and our finding that the SL biosynthetic pathway is critical for Shiga toxin sensitivity in HeLa cells (Fig. 1*B*). *LAPTM4A* and *TM9SF2*, which have been correlated with Shiga toxin resistance (25–27), were also found to be highly enriched and among the 20 top-ranking genes in our genetic screen. Surprisingly, AHR, a transcription factor with broad physiological functions (14–19), also showed high enrichment comparable with that observed for the SL biosynthetic genes (Fig. 1*F*).

### AHR is a positive regulator of SL levels in HeLa cells

**Figure 2.**
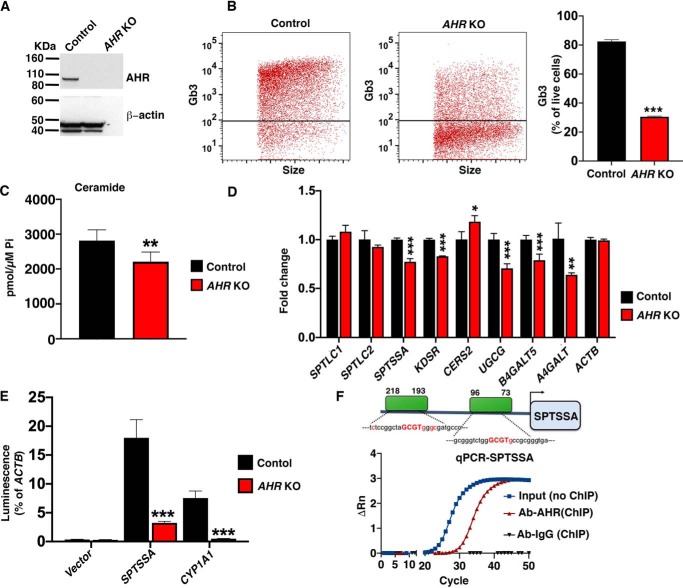
**AHR is a positive regulator of SL levels in HeLa cells.** *A*, representative immunoblot analysis of AHR protein expression in WT (control) and *AHR* KO HeLa cells. β-Actin was used as a loading control. *B*, flow cytometry analysis of WT (control) and *AHR* KO HeLa cells stained with anti-CD77 antibody. *Left*, representative dot plot of cell-surface expression of Gb3. *Right*, quantification of Gb3 cell-surface expression (mean ± S.D. (*error bars*); *n* = 3). Unpaired *t* test; *** *p* ≤ 0.001. *C*, levels of total ceramide determined by HPLC-tandem MS on lipid extracts from WT (control) and *AHR* KO HeLa cells. Data are expressed as mean ± S.D. Unpaired Student's *t* test; **, *p* ≤ 0.01. *n* = 5 for control cells; *n* = 7 for *AHR* KO cells. *D*, RT-qPCR of SL biosynthetic genes in WT (control) and *AHR* KO HeLa cells. Probes detect genes as labeled. Data are expressed as mean ± S.D., normalized to transcript level of *ACTB*. Values from control were set at 1.0. Unpaired *t* test was used: *, *p* ≤ 0.05; **, *p* ≤ 0.01; ***, *p* ≤ 0.001. *n* = 4 for each genotype. *E*, vectors containing the promoters of *SPTSSA*, *CYP1A1*, or *ACTB* were transfected into WT (control) or *AHR* KO HeLa cells, and luciferase assays were conducted 48 h later. Data are expressed as mean ± S.D., normalized to the *ACTB* promoter activity. Unpaired *t* test was used: ***, *p* ≤ 0.001. *F*, *top*, *schematic representation* of AHR-binding sites in the promoter region of *SPTSSA* predicted by Genomatix analysis. *Bottom*, ChIP with anti-AHR antibody followed by qPCR with probes designed against the *SPTSSA* promoter region containing predicted AHR-binding sites. ChIP with mouse IgG was used as a negative control.

To directly determine whether AHR regulates SL levels, an *AHR* KO HeLa cell line was generated by Cas9-mediated disruption of the *AHR* gene (Fig. 2*A*). Compared with control cells, Gb3 cell-surface expression was significantly reduced in *AHR* KO HeLa cells (Fig. 2*B*). Ceramide levels, both for some individual species (Fig. S1) and total ceramide (Fig. 2*C*), were significantly decreased in *AHR* KO cells compared with control HeLa cells. The mRNA expression of SL biosynthetic pathway genes *SPTSSA*, *KDSR*, *UGCG*, *B4GALT5*, and *A4GALT* was significantly reduced in the absence of *AHR,* whereas *CERS2* expression was enhanced (Fig. 2*D*). These results indicate that *AHR* is needed for normal expression of several key genes in the Gb3 biosynthesis pathway and that SL levels, both ceramide and Gb3, are reduced in the absence of *AHR*.

*SPTSSA* encodes a subunit of SPT, the rate-limiting enzyme in the SL biosynthetic pathway (Fig. 1*A*) (30). Because the reduced mRNA expression of *SPTSSA* could contribute to the lower ceramide and Gb3 levels in *AHR* KO HeLa cells, we next determined whether AHR was required for maximal *SPTSSA* promoter activity. A reporter plasmid construct was prepared containing ∼1 kb of genomic sequence upstream of the transcriptional start site of *SPTSSA* fused to the luciferase gene. We used a luciferase plasmid without a promotor sequence inserted (empty vector) and a luciferase reporter plasmid containing the promoter of the *CYP1A1* gene, which is known to be activated by AHR (14, 31), as controls. The plasmids were transfected into either control HeLa cells or *AHR* KO HeLa cells. After 48 h, luciferase activity normalized to actin promoter activity in each cell type was determined. Whereas the empty vector control showed little promoter activity in either cell type, both the *SPTSSA* and *CYP1A1* promoters induced luciferase activity in control HeLa cells. Significantly less luciferase activity was detected for both promoters in *AHR* KO cells, indicating that AHR expression was needed for full activities of these promoters in HeLa cells (Fig. 2*E*).

ChIP-quantitative PCR (qPCR) (32) was used to determine whether AHR interacted directly with the promoter region of *SPTSSA*, which contains two putative AHR-binding motifs (Fig. 2*F*). AHR antibody specifically immunoprecipitated the *SPTSSA* promotor sequences from HeLa cell sheared chromatin as determined by PCR amplification. The *SPTSSA* promotor sequences were not PCR-amplified after immunoprecipitation of HeLa cell sheared chromatin with a control IgG (Fig. 2*E*). These results suggest that AHR binds directly to the *SPTSSA* promoter.

### AHR is a positive regulator of SL levels in mice

**Figure 3.**
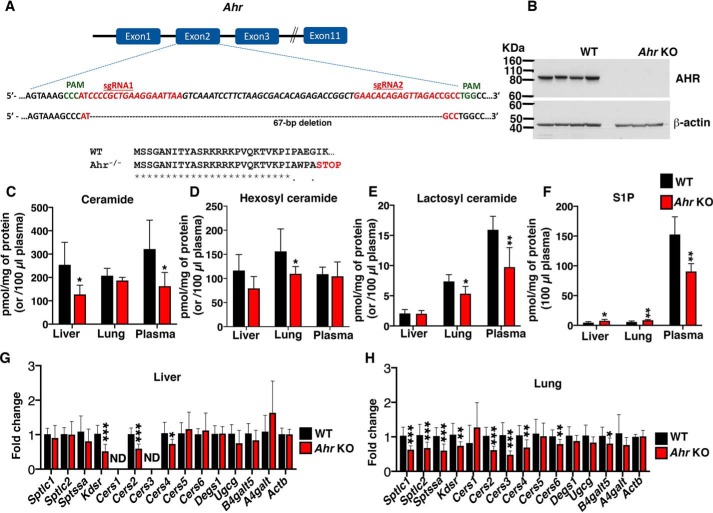
**AHR is a positive regulator of SL levels in mice.** *A*, *schematic* illustrates the first three and the last intron-exon organization of the *Ahr* gene. *Ahr* KO mice were produced by CRISPR/Cas9-induced mutations resulting in a frameshift and premature stop codon. The locations of sgRNA target sequences and PAM sites (*green*) and the alignment of amino acid sequences, showing changes introducing a premature stop codon in the *Ahr* KO mice, are noted. *B*, representative immunoblot analysis of AHR expression in lung tissue from 5-week-old WT and *Ahr* KO mice. β-Actin was used as a loading control. *C–F*, levels of total ceramide, hexosyl ceramide, lactosyl ceramide, and S1P in tissues (liver, lung, and plasma) from 5-week-old WT and *Ahr* KO mice. Data are expressed as mean ± S.D. (*error bars*). Unpaired *t* test was used: *, *p* ≤ 0.05. For liver and lung, *n* = 6 for each genotype. For plasma, *n* = 6 for WT mice and *n* = 5 for *Ahr* KO mice. *G* and *H*, RT-qPCR of SL biosynthetic genes in liver and lung tissues from 5-week-old WT and *Ahr* KO mice. Probes detect gene sequences as *labeled*. Data are expressed as mean ± S.D., normalized to transcript level of *ACTB*. Values from control were set at 1.0. Unpaired *t* test was used: *, *p* ≤ 0.05; **, *p* ≤ 0.01; ***, *p* ≤ 0.001. For liver, *n* = 4 for WT mice and *n* = 3 for *Ahr* KO mice. For lung, *n* = 4 for each genotype. *ND*, not detectable.

To determine whether AHR has a physiologic role in regulating SL levels, we established *Ahr* KO mice by Cas9-mediated gene disruption (33). Mice carrying a null *Ahr* allele were generated by injection of C57BL/6J embryos with two sgRNAs targeting *Ahr* exon 2, which is the first protein-coding exon (Fig. 3*A*). The Cas9-mediated targeting resulted in a 67-bp deletion that is predicted to cause a premature stop codon. Immunoblot analysis from homogenates of lung tissue, where *Ahr* is highly expressed (34), showed undetectable AHR protein expression in the *Ahr* KO mice compared with robust expression from WT controls (Fig. 3*B*).

LC-MS analysis of liver tissue extracts showed a significant decrease in some individual ceramide species (Fig. S2) and total ceramide levels (Fig. 3*C*) in *Ahr* KO mice compared with WT mice. No significant changes were observed in either total hexosyl (Fig. 3*D*) or lactosyl ceramide (Fig. 3*E*) levels in liver extracts from *Ahr* KO mice. In lungs, levels of some low-abundance ceramide species were significantly reduced in *Ahr* KO mice (Fig. S3), but no significant change was observed in total ceramide levels in *Ahr* KO mice (Fig. 3*C*); however, both total hexosyl and lactosyl ceramide levels were significantly decreased in *Ahr* KO mice compared with WT littermates (Fig. 3, *D* and *E*). Plasma, which has much of its SLs supplied by liver (35), had a significant decrease in both some individual species of ceramide (Fig. S4) and total ceramide levels (Fig. 3*C*) No change was observed in total hexosyl ceramide levels, but lactosyl ceramide levels were significantly decreased (Fig. 3, *D* and *E*) in *Ahr* KO mice compared with WT controls. Levels of S1P, a signaling SL metabolite, were significantly reduced in *Ahr* KO plasma compared with that observed in WT plasma (Fig. 3*F*). Liver and lung S1P levels, which are relatively low compared with plasma levels, were significantly elevated in the *Ahr* KO mice (Fig. 3*F*).

RT-qPCR analysis of a panel of SL biosynthetic genes in *Ahr* KO and WT mouse tissues demonstrated that mRNA levels of the *Kdsr*, *Cers2*, and *Cers4* genes were significantly reduced in the liver of *Ahr* KO compared with WT mice. In lungs, mRNA levels of 8 of the 14 SL biosynthetic genes tested were significantly lower in the *Ahr* KO mice compared with WT mice (Fig. 3, *G* and *H*). These results indicate that AHR positively regulates the expression of sphingolipid biosynthetic genes and sphingolipid levels in mice.

### AHR deficiency reduces SL levels and myelin thickness in sciatic nerve

**Figure 4.**
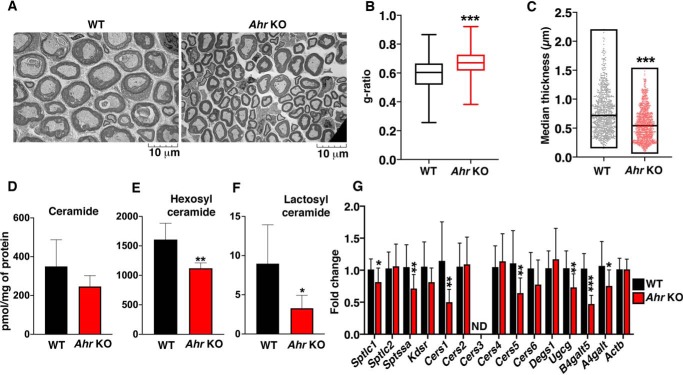
**AHR deficiency reduces SL levels and myelin thickness in sciatic nerve.** *A*, representative transmission EM images of sciatic nerve from 3-week-old WT and *Ahr* KO mice. *B*, g-ratios for 3-week-old WT and *Ahr* KO mice sciatic nerve. Data are expressed as mean ± S.D. (*error bars*). Unpaired *t* test was used: ***, *p* ≤ 0.001. *n* = 3 for WT mice; *n* = 4 for *Ahr* KO mice. *C*, median myelin thickness of axons in 3-week-old WT and *Ahr* KO mice. *n* = 3 for WT mice; *n* = 4 for *Ahr* KO mice. Statistical significance was determined by nonparametric Mann–Whitney test; ***, *p* ≤ 0.001. *D–F*, levels of total ceramide, hexosyl ceramide, and lactosyl ceramide in sciatic nerve from 5-week-old WT and *Ahr* KO mice. Data are expressed as mean ± S.D. Unpaired *t* test was used: *, *p* ≤ 0.05. *n* = 6 for each genotype. *G*, RT-qPCR of SL biosynthetic genes in sciatic nerve from 3-week-old WT and *Ahr* KO mice. Probes detect gene sequences as *labeled*. Data are expressed as mean ± S.D., normalized to transcript level of *ACTB*. Values from control were set at 1.0. Unpaired *t* test was used: *, *p* ≤ 0.05; **, *p* ≤ 0.01; ***, *p* ≤ 0.001. *n* = 4 for each genotype. *ND*, not detectable.

*Ahr* deficiency in mice has been reported to cause abnormally thin myelin sheaths during development (36, 37). Myelin formation is critically dependent on SL production, where SLs constitute the majority of nonsterol lipid in the expansive myelin membrane (12, 38). To evaluate myelination in our *Ahr* KO mice, we performed ultrastructural analysis of actively myelinating sciatic nerves (Fig. 4*A*). Myelinated axons in sciatic nerves of *Ahr* KO mice had a significantly higher g-ratio (the ratio of the inner axonal diameter to the total outer diameter) compared with WT mice, which is indicative of thinner myelin (Fig. 4*B*). A reduction in median myelin thickness in *Ahr* KO sciatic nerve compared with that in controls was confirmed by direct measurements (Fig. 4*C*).

LC-MS analysis of lipid extracts of sciatic nerves from *Ahr* KO mice showed a significant decrease in levels of the major ceramide long-chain fatty acid species (C24; Fig. S5), as well as hexosyl (Fig. 4*E*) and lactosyl (Fig. 4*F*) ceramide, compared with those of WT mice. Total ceramide was not significantly different between WT and *Ahr* KO sciatic nerve (Fig. 4*D*). Determination of mRNA levels of genes in the SL biosynthetic pathway in sciatic nerve revealed significantly decreased mRNA levels of *Sptlc1*, *Sptssa*, *Cers1*, *Cers5*, *Ugcg*, *B4galt5*, and *A4galt* in *Ahr* KO mice relative to WT mice (Fig. 4*G*). These observations show that AHR is required for full myelination of the sciatic nerve axons, as well as normal mRNA expression of genes in the SL biosynthetic pathway and regulation of SL levels in this tissue.

## Discussion

In a loss-of-function CRISPR/Cas9 genetic screen targeting positive regulators of membrane SLs in HeLa cells, we identified the ligand-activated transcription factor AHR. Recently, similar Shiga toxin-based genome-wide screens have been reported in various cell types (25–27). As in our screen, they have identified genes in the SL biosynthetic pathway and other genes that regulate GB3 expression, notably LAPTMA and TM9SF2. Our screen was unique in the identification of AHR.

We showed that AHR expression was needed for full mRNA expression of several key genes in the SL biosynthesis pathway in HeLa cells and mouse tissues, consistent with our finding of decreased SL content in both HeLa cells and mouse tissues in the absence of AHR expression. In actively myelinating axons of the sciatic nerve, we found that AHR is needed to produce the full thickness of the SL-rich myelin sheath. Collectively, these results support the conclusion that AHR is a physiologically important positive regulator of SL levels. Importantly, AHR had previously been shown to be a regulator of the synthesis of other classes of lipids, including sterols, fatty acids, and glycerolipids (39, 40).

AHR is a basic-helix-loop-helix, Per-Arnt-Sim transcription factor that is activated by small molecules derived from the diet, the microbiome, endogenous metabolism, and the environment (41, 42). AHR exists in the cytosol as an inactive complex and, upon ligand binding, translocates to the nucleus and heterodimerizes with aryl hydrocarbon receptor nuclear translocator (ARNT). This heterodimer acts as a transcription factor on promoters containing dioxin/xenobiotic response elements (43). Whereas AHR has been well-established as an important sensor of environmental xenobiotics, it is now appreciated that the transcriptional activity of AHR is also important in development and the functioning of several physiological systems (14, 15).

AHR agonists from endogenous and dietary sources include metabolites of arachidonic acid, heme, tryptophan, indole, and flavonoids (44). The influence of AHR activity on sphingolipid levels suggests that sphingolipid metabolism may be regulated by one or more of these AHR ligands, an area for future investigation.

Myelin is ∼80% lipid by dry weight, with much of the nonsterol lipid made up of SLs (38). The formation of the growing Schwann cell myelin membrane, which expands several thousand-fold during development, requires an extremely high influx of new substrate produced through the SL biosynthetic pathway, mostly in the form of hexosyl ceramide (galactosyl ceramide) (45). Previous results have shown that *Ahr* KO mice have thinner myelin sheaths, dysregulated myelin gene expression, and locomotor deficiencies (36, 37). We confirmed this abnormally thin myelination and have now shown that some SL gene expression levels, as well as the hexosyl ceramide levels that are critical for myelin formation, are significantly reduced in actively myelinating sciatic nerves of *Ahr* KO mice. Dietary sphingolipids have been shown to contribute to CNS myelination under conditions of low SPT activity in developing rats (46). Thus, the extent of myelination in the AHR KO mice may be affected by the levels of sphingolipids in the diet.

AHR has also been shown to mediate homeostasis in skin, a tissue whose development and function is critically dependent on the SL biosynthesis pathway (19). Mice with a keratinocyte-specific deficiency of ARNT, the AHR binding partner, exhibit severe skin barrier dysfunction and die because of rapid dehydration (47). In addition, these mice have impaired gene expression of an isozyme of dihydroceramide desaturase (*Degs*), a gene in the *de novo* ceramide pathway (Fig. 1*A*), along with changes in the ceramide composition of the epidermis. An *Ahr* KO mouse has also been reported to have a disrupted skin permeability barrier (19), although not as severe as that observed in the *Arnt* KO mouse model, suggesting that ARNT and AHR may have partially divergent functions in skin. S1P is a bioactive lipid found in relatively high amounts in the circulation (48). By signaling through a family of G protein–coupled receptors, it has important functions in the development and functioning of the immune and vascular systems, which are also regulated by AHR (14, 48). The significant reduction of plasma S1P levels observed in the *Ahr* KO mouse suggests that AHR regulation of circulating S1P levels may contribute to AHR's role in the immune and vascular systems.

In conclusion, we have found that AHR is a positive regulator of SL levels, presumably through enhancing mRNA expression of several key genes in the SL biosynthetic pathway. We also demonstrated that AHR regulation is important in the context of myelination, a process that requires high levels of membrane SLs. The positive regulation of SL levels may also play roles in the diverse functions mediated by AHR.

## Experimental procedures

### Genome-wide CRISPR screen

The genome-wide CRISPR screen was performed as described (49). Human GeCKOv2 CRISPR knockout pooled library was a gift from Feng Zhang (Addgene, catalog no. 1000000049). HeLa cells expressing Cas9 (5.6 × 10^7^) were transduced with the GeCKOv2 lentivirus library (28, 49) to achieve a total coverage of ∼55× with an MOI of 0.25. Puromycin (Thermo Fisher Scientific, catalog no. A1113803) (2 μg/ml) was added 24 h post-transduction. After 72 h of puromycin selection, cells were collected and divided into two groups (2.8 × 10^7^ cells/group) and cultured for 2 weeks in either the presence (resistant) or absence (control) of 2 ng/ml Shiga toxin (List Biological Laboratories). After toxin treatment, genomic DNA was extracted from cells using the Blood and Cell Culture Midi Kit (Qiagen, catalog no. 13343).

Genomic DNA (130 μg) from each group was used as a template DNA for the PCR to amplify the sgRNA region. Separate (13 × 100 μl) reactions with 10 μg of genomic DNA were set up using NEBNext Ultra^TM^ II Q5 master mix for PCR (New England Biolabs, catalog no. M0544L) with the following primer set: forward, 5′-AATGGACTATCATATGCTTACCGTAACTTGAAAGTATTTCG; reverse, 5′-CTTTAGTTTGTATGTCTGTTGCTATTATGTCTACTATTCTTTCCA.

PCR product from the first round of PCRs (5 μl) was used as a template for the second round PCR to amplify and attach Illumina-compatible multiplexing sequencing adopters using the same PCR conditions. Finally, PCR products were purified and quantified. Barcoded PCR amplicons were subjected to Illumina platform–based next-generation sequencing.

### Animal model: Ahr KO mice

All animal procedures were approved by the NIDDK, National Institutes of Health, Animal Care and Use Committee and were performed in accordance with National Institutes of Health guidelines. The C57BL/6J (Jackson Laboratory) strain was used as an embryo donor. Fertilized oocytes were injected with 100 ng/μl Cas9 mRNA and 50 ng/μl each sgRNA (Synthego) (Fig. 3*A*). The injected oocytes were transferred to the oviducts of pseudopregnant female mice. Tails from 21-day-old progeny mice were genotyped using forward primer (5′-GTTTTTCTAGGTAAAATTTGCTTTAAAATCGTT) and reverse primer (5′-GGAAAGTTAGTAACAGTTACACATTTAGCTTTG) with PCR conditions as follows: 94 °C for 10 min, 94 °C for 30 s, 64 °C for 15 s, 68 °C for 30 s for 40 cycles. WT allele yielded a 722 bp band, and the KO allele yielded a 655 bp band.

Sciatic nerves harvested from 3-week-old WT and *Ahr* KO mice were fixed in 2% paraformaldehyde, 2.5% glutaraldehyde, and 0.1 m cacodylate buffer. Sciatic nerves were fixed with 1% glutaraldehyde, 4% paraformaldehyde at 4 °C for 24 h. Ultrathin cross-sections were prepared and analyzed with an electron microscope (Philips EM410). g-ratios were calculated by measuring the mean g-ratio of all of the myelinated axons in three independent microscopic fields with 1.25 × 10^3^ magnification.

### Statistical analysis

Statistical analysis was performed using GraphPad Prism (version 8.2.1). Significance was determined by unpaired *t* test, with α = 0.05.

## Author contributions

S. M., C. L., G. T., and R. L. P. conceptualization; S. M., M. K., Y. T. L., C. B., G. T., and R. L. P. investigation; S. M., M. K., C. B., C. L., G. T., and R. L. P. writing-original draft; S. M., M. K., Y. T. L., C. B., C. L., G. T., and R. L. P. writing-review and editing; Y. T. L., C. B., C. L., G. T., and R. L. P. methodology; C. L., G. T., and R. L. P. resources; C. L., G. T., and R. L. P. supervision.

## Supplementary Material

Supporting Information

## Footnotes

2 The abbreviations used are:

SL: sphingolipid
AHR: aryl hydrocarbon receptor
ARNT: aryl hydrocarbon receptor nuclear translocator
Gb3: globotriaosylceramide
GeCKO: genome-scale CRISPR/Cas9 knockout
KO: knockout
MOI: multiplicity of infection
qPCR: quantitative PCR
S1P: sphingosine-1-phosphate
SPT: serine palmitoyltransferase
sgRNA: single guide RNA.
