# Supplementary material for: A genome-wide CRISPR/Cas9 screen reveals that the aryl hydrocarbon receptor stimulates sphingolipid levels
Source: J Biol Chem. 2020 Feb 6;295(13):4341–9. doi: 10.1074/jbc.AC119.011170 (PMC7105297; doi:10.1074/jbc.AC119.011170)
Supplement: Supporting Information [file supp_295_13_4341__index.html]

A genome-wide CRISPR/Cas9 screen reveals that the aryl hydrocarbon receptor stimulates sphingolipid levels — Aryl hydrocarbon receptor regulates sphingolipid levels — A genome-wide CRISPR/Cas9 screen reveals that the aryl hydrocarbon receptor stimulates sphingolipid levels — ACCELERATED COMMUNICATION: AHR regulates sphingolipids — Supporting Information 

# A genome-wide CRISPR/Cas9 screen reveals that the aryl hydrocarbon receptor stimulates sphingolipid levels

## Supporting Information

- Supporting Information (to be published online) - Supplemental Figures S1-S5 Supplemental Tables S1-S4
