# Supplementary material for: A genome-wide CRISPR/Cas9 screen reveals that the aryl hydrocarbon receptor stimulates sphingolipid levels
Source: J Biol Chem. 2020 Feb 6;295(13):4341–9. doi: 10.1074/jbc.AC119.011170 (PMC7105297; doi:10.1074/jbc.AC119.011170)
Supplement: Supporting Information [file supp_AC119.011170_155985_1_supp_470744_q5ccbm.pdf]

A genome-wide CRISPR/Cas9 screen reveals that the aryl hydrocarbon receptor stimulates sphingolipid levels

Saurav Majumder, Mari Kono, Y. Terry Lee, Colleen Byrnes, Cuiling Li, Galina Tuymetova, and Richard L. Proia

From the Genetics of Development and Disease Branch, National Institutes of Health, Bethesda, MD, 20892

Supporting Information

Supplemental Figures S1-S7
Supplemental Tables S1-S4
Supporting Experimental Procedures

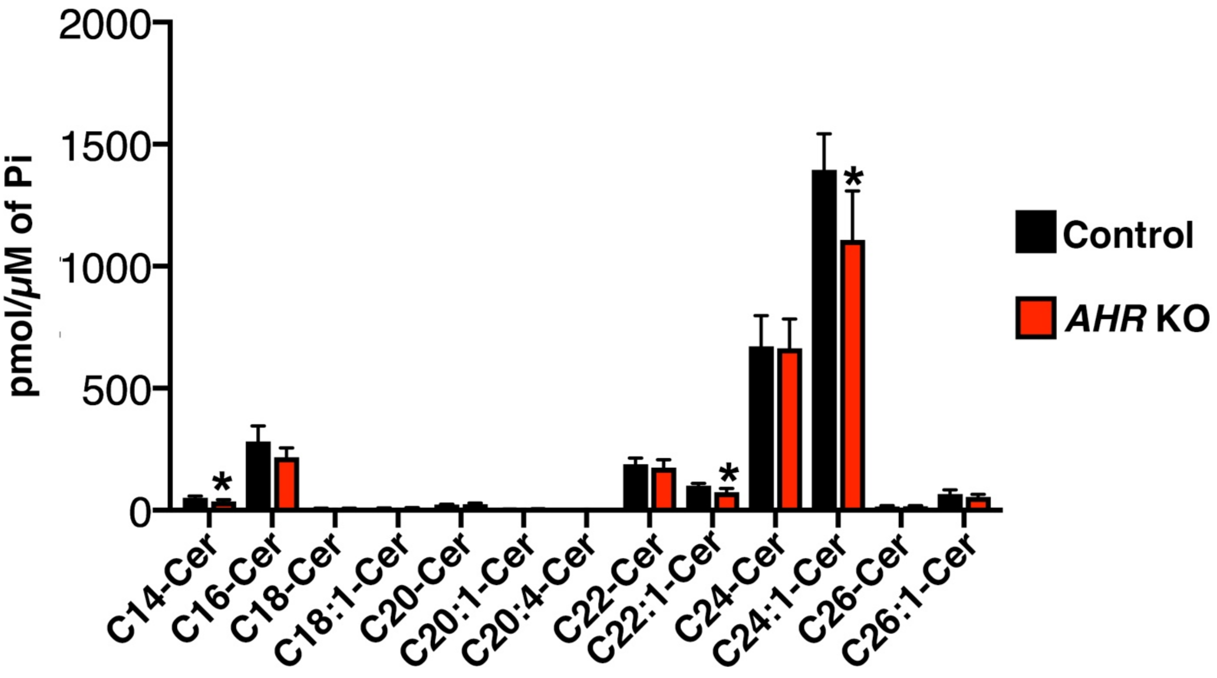

Figure S1. Levels of individual ceramide subspecies with different fatty-acid chain lengths determined by HPLC-tandem MS on lipid extracts from WT (control) and *AHR* KO HeLa cells. Data are expressed as mean ± SD. Unpaired t test; * p≤0.05. n=5 for control cells; n=7 for *AHR* KO cells.

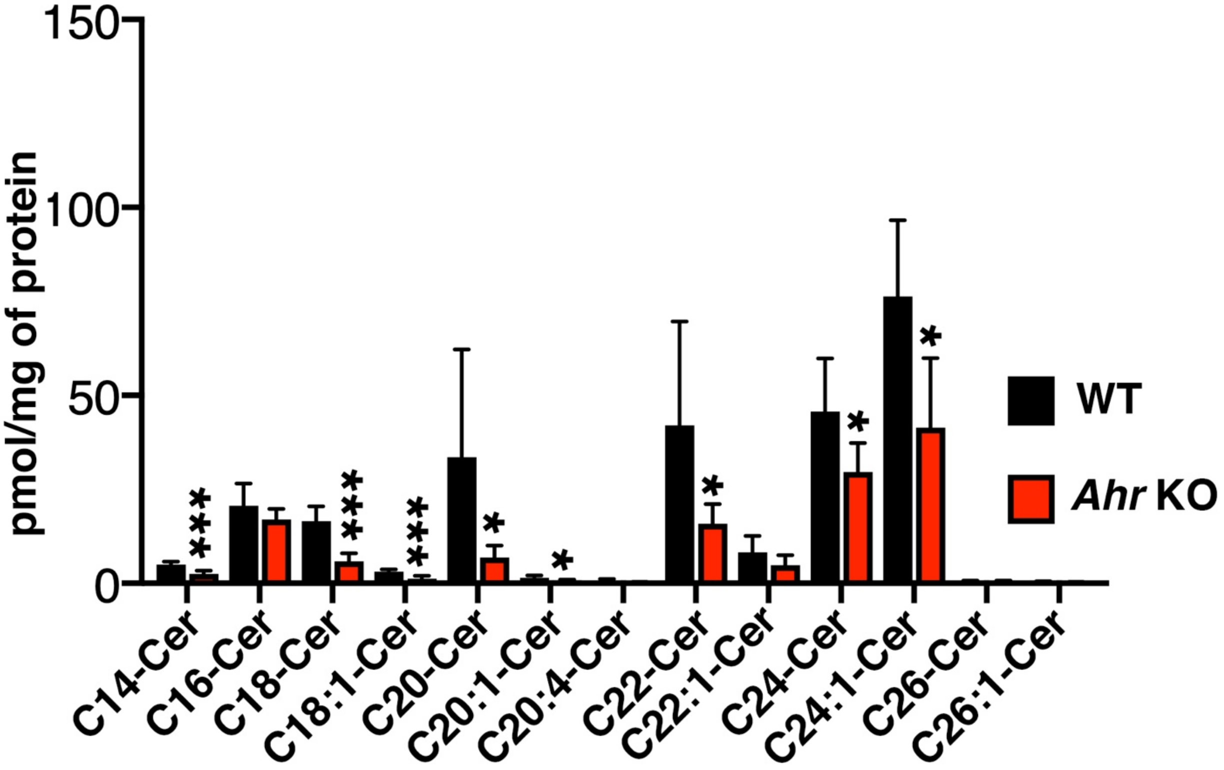

Figure S2. Levels of individual ceramide subspecies with different fatty-acid chain lengths determined by HPLC-tandem MS on lipid extracts of liver harvested from 5-week-old WT or *Ahr* KO mice. Data are expressed as mean ± SD. Unpaired t test; * p≤0.05, *** p≤0.001. n=6 for both genotypes.

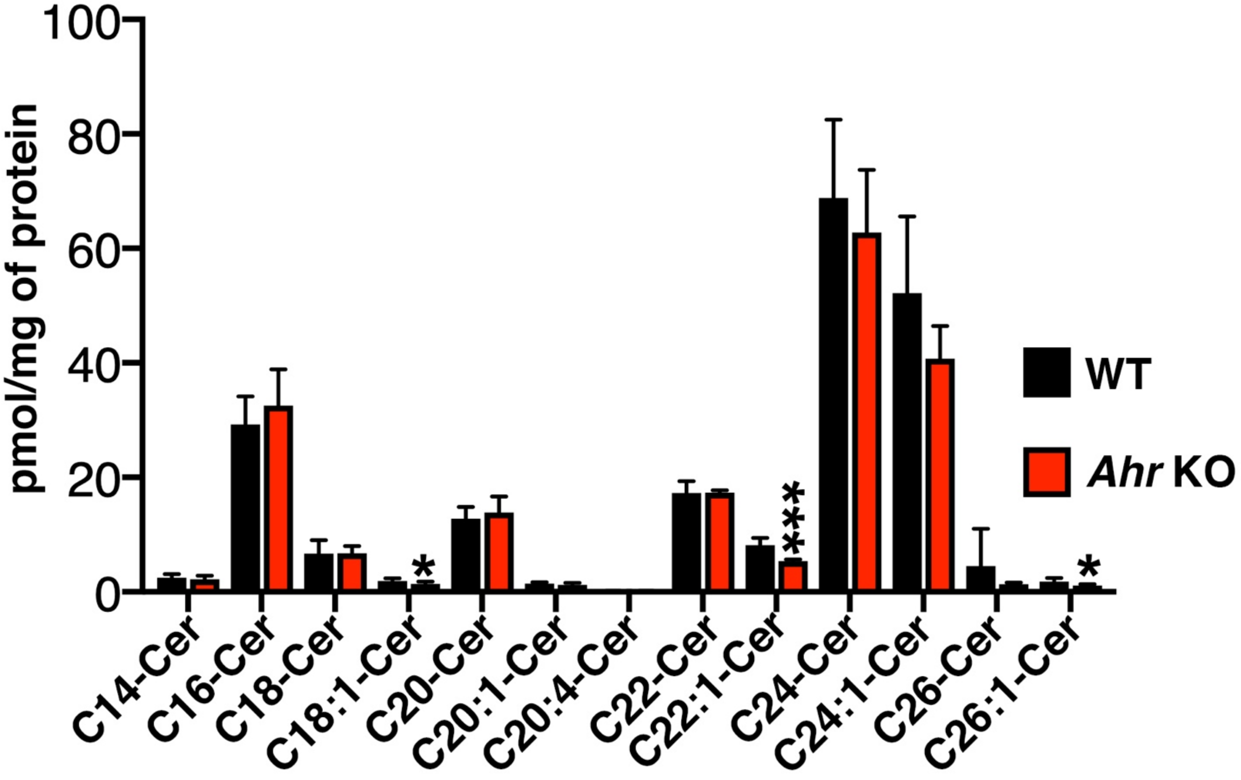

Figure S3. Levels of individual ceramide subspecies with different fatty-acid chain lengths determined by HPLC-tandem MS on lipid extracts of lung harvested from 5-week-old WT or *Ahr* KO mice. Data are expressed as mean ± SD. Unpaired Student's t test; * p≤0.05, *** p≤0.001. n=6 for both genotypes.

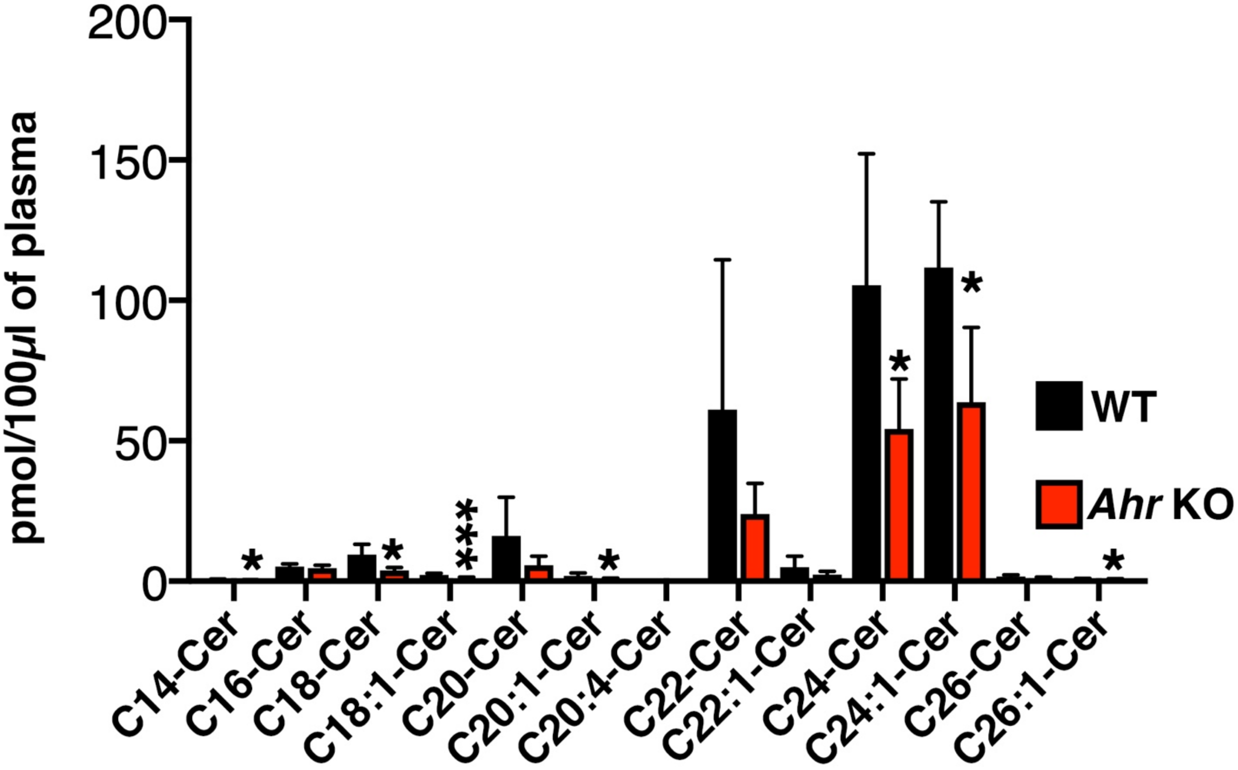

Figure S4. Levels of individual ceramide subspecies with different fatty-acid chain lengths determined by HPLC-tandem MS on lipid extracts of plasma harvested from 5-week-old WT or *Ahr* KO mice. Data are expressed as mean ± SD. Unpaired Student's *t* test; * p≤0.05, *** p≤0.001. n=6 for WT mice; n=5 for *Ahr* KO mice.

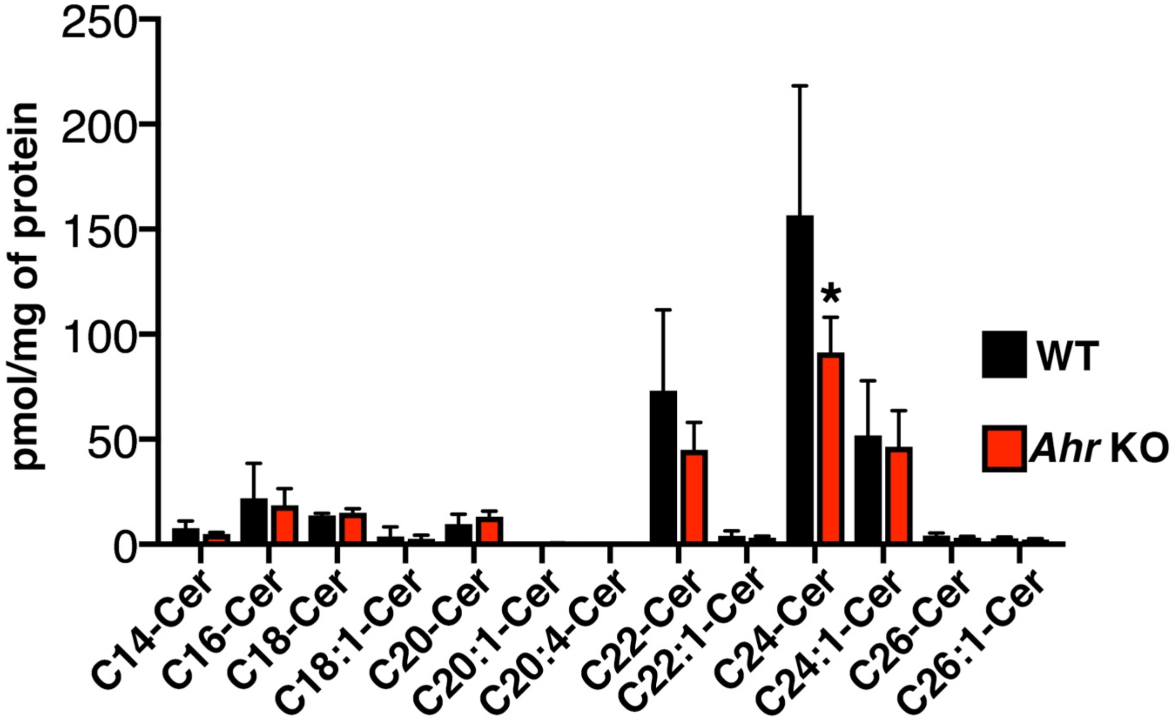

Figure S5. Levels of individual ceramide subspecies with different fatty-acid chain lengths determined by HPLC-tandem MS on lipid extracts of sciatic nerve harvested from 5-week-old WT or *Ahr* KO mice. Data are expressed as mean ± SD. Unpaired Student's t test; * $p \leq 0.05$. n=6 for WT mice; n=5 for *Ahr* KO mice.

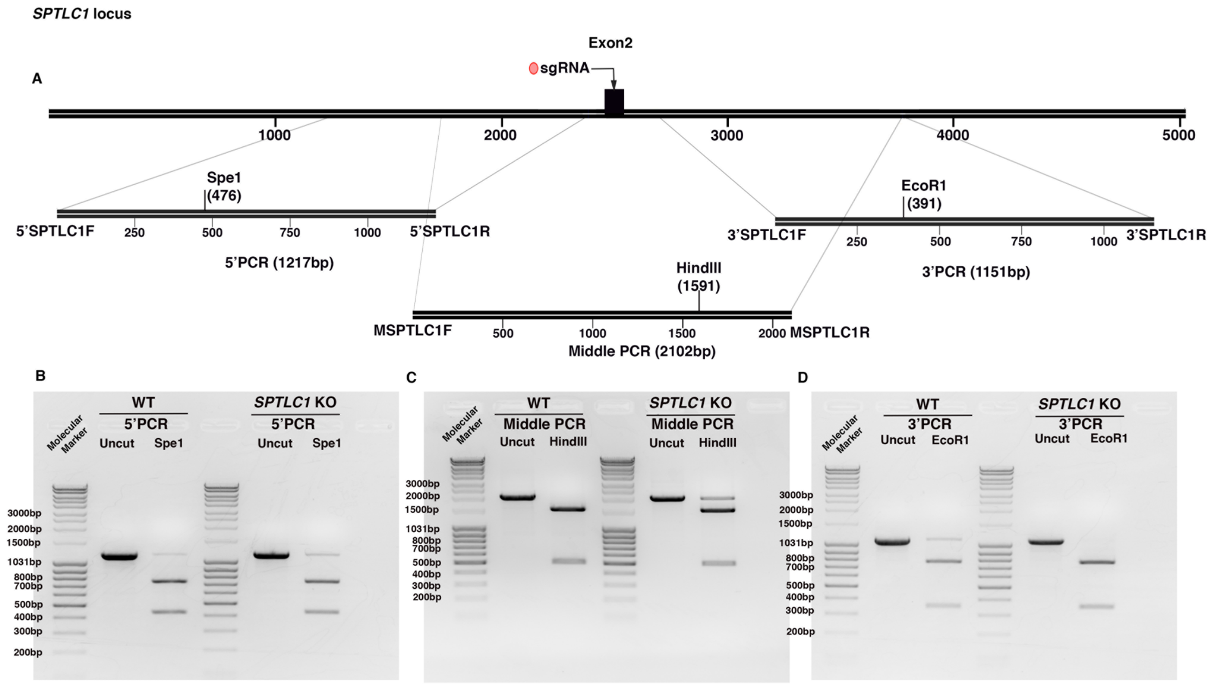

Figure S6. No gross alterations in the *SPTLC1* locus in *SPTCL1* KO HeLa cells. (A) Upper scheme, 5kb segment of intron-exon organization of *AHR* centering on the sgRNA binding site. Lower scheme, representation of individual PCR products (5'PCR, middle PCR, 3'PCR) with location of unique restriction sites of Spe1, HindIII and EcoR1 (above) and PCR primer names (below). (B-D) PCR products of the targeted regions from the genomic DNA of both wild type (parental) and *AHR* KO cells were digested by the restriction enzymes. Uncut and restriction digests were separated in a 1.2% agarose gel.

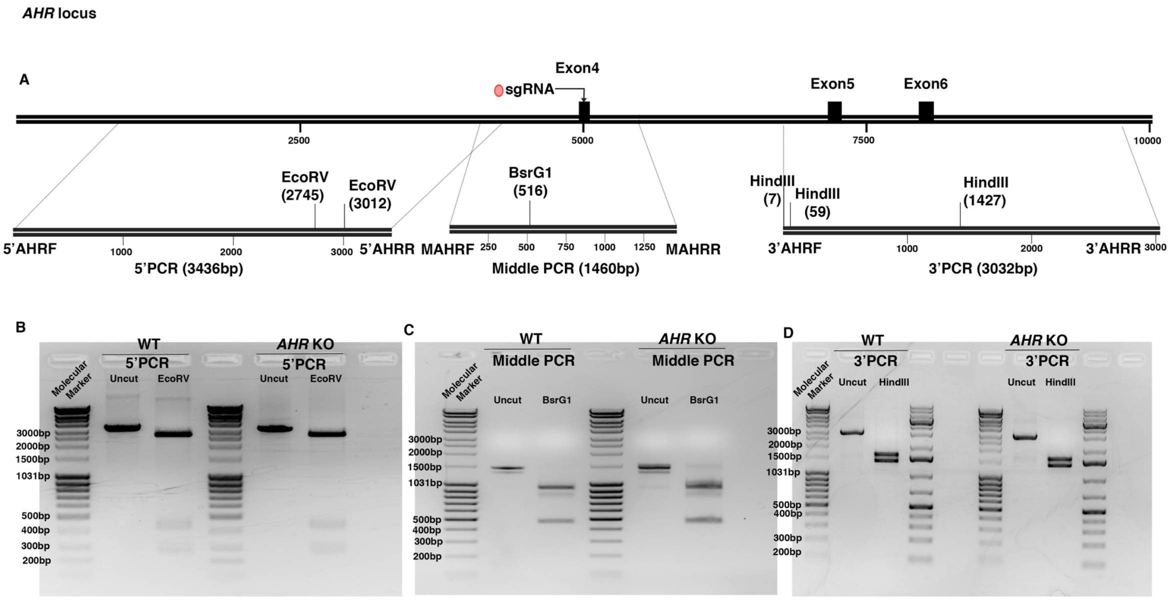

Figure S7. No gross alterations in the *AHR* locus in *AHR* KO HeLa cells. (A) Upper scheme, 10kb segment of intron-exon organization of *AHR* centering on the sgRNA binding site. Lower scheme, representation of individual PCR products (5'PCR, middle PCR, 3'PCR) with location of unique restriction sites of EcoRV, BsrG1 and HindIII (above) PCR primer names (below). (B-D) PCR products of the targeted regions from the genomic DNA of both wild type (parental) and *AHR* KO cells were digested by the restriction enzymes. Uncut and restriction digests were separated in a 1.2% agarose gel.

id	num	pos|score	pos|rank	pos|lfc
LAPTM4A	6	2.04E-18	1	8.7732
GOLPH3	6	5.45E-14	2	5.2029
TM9SF2	6	1.20E-13	3	9.3209
SLC35A2	6	4.79E-13	4	8.9964
ARPC3	6	2.04E-12	5	6.7625
SPTSSA	6	3.90E-12	6	7.8078
UGCG	6	6.92E-12	7	8.401
ACTR2	6	4.41E-11	8	7.2859
B4GALT5	6	5.92E-11	9	8.0147
SPTLC2	6	2.40E-09	10	5.6465
ACTR3	6	9.25E-09	11	4.7234
A4GALT	6	2.98E-08	12	4.4771
AHR	6	6.29E-08	13	3.5972
ARPC4	6	5.24E-07	14	3.7674
UGP2	6	1.36E-06	15	3.436
SPTLC1	6	3.00E-06	16	1.0891
VPS53	6	4.72E-06	17	1.3603
COG4	6	5.48E-06	18	2.1785
TMEM165	6	8.01E-06	19	2.0017
NCKAP1	6	1.12E-05	20	1.0593
NPY2R	6	1.31E-05	21	1.8482
MOGS	6	1.38E-05	22	1.0496
COG3	6	2.01E-05	23	1.7232
SPPL3	6	2.16E-05	24	1.541
RAC1	6	2.31E-05	25	-0.96525
VPS51	6	2.37E-05	26	1.8331
CWC22	6	2.51E-05	27	-1.0452
SMARCB1	6	2.65E-05	28	1.1984
COG8	6	4.16E-05	29	-0.019937
SPCS3	6	7.53E-05	30	-1.0452
GLTP	6	0.00011743	31	-2.7948
SLC25A21	6	0.0001755	32	-5.0992
CCDC37	6	0.00017578	33	-1.0452
TMEM40	6	0.00022599	34	-2.4677
IQCF1	6	0.00027621	35	-5.2565
ARNT	6	0.00029123	36	-0.13814
PLIN1	6	0.00033542	37	-1.0452
COMMD3	6	0.0004094	38	-0.51101
TLX1	6	0.00042684	39	-3.2691
FBXL14	6	0.00042771	40	-1.0452
CERS2	6	0.00045047	41	1.2233
FAM162B	6	0.00046689	42	-1.0452
C5orf63	6	0.00052725	43	-2.6888
C14orf132	6	0.00054732	44	-1.0452
ANKRD28	6	0.00057746	45	-2.4363
FGFR1	6	0.00057987	46	-2.0617
IL22	6	0.00061809	47	-1.0452
GCSAML	6	0.00062766	48	-2.7948
PTH1R	6	0.00070655	49	-2.6239
hsa-mir-3158-1	4	0.00071046	50	-0.72495
SYP	6	0.00072805	51	-2.0617
PER3	6	0.00073387	52	-0.56582
C19orf38	6	0.00074425	53	-2.8688
STIP1	6	0.00078826	54	0.199
SLC22A20	6	0.00082844	55	-3.8304
GALE	6	0.00084086	56	-0.55601
hsa-mir-4539	4	0.00085356	57	-1.0452
FBLN5	6	0.00085845	58	-1.0452
PTPN11	6	0.00085845	59	-0.56582
GPR161	6	0.00085845	60	-4.5279
KCNA2	6	0.00085845	61	-1.0452
NLRP6	6	0.00087863	62	-2.0617
DSG1	6	0.00092881	63	-5.0826
C1orf56	6	0.000979	64	-1.0452
ATXN7L3B	6	0.0010106	65	-2.4677
DUS4L	6	0.0010292	66	-3.2748
RBM12B	6	0.0010658	67	-3.1431
ARPC2	6	0.0010794	68	-2.3228
IMMP2L	6	0.0011097	69	-1.9817
DENND1B	6	0.0011295	70	-5.5882
ANKRD23	6	0.0011437	71	-2.035
SORBS3	6	0.0011695	72	-3.1607
IWS1	6	0.0011825	73	-3.7784
OST4	5	0.0011933	74	-5.7104
hsa-mir-30c-1	4	0.001322	75	-2.0027
KLF4	6	0.0013302	76	-3.1403
RHEB	6	0.0013305	77	-1.0452
PRR15	6	0.0013629	78	-2.0672
HS2ST1	6	0.0013769	79	-2.5176
RIN2	6	0.0013804	80	-6.1281
UXS1	6	0.0014242	81	0.17014
DLX5	6	0.001429	82	-2.414
RMDN1	6	0.0014306	83	-4.0942
ACTL7A	6	0.0014554	84	-5.4307
PTPN6	6	0.0015882	85	-2.414
ELF3	6	0.0016084	86	-0.31197
COG6	6	0.001667	87	-1.2126
KRTAP9-9	6	0.0016814	88	-1.0452
LTF	6	0.0017176	89	-2.414
hsa-mir-5579	4	0.0017567	90	0.15385
MAGED4	6	0.0017817	91	-4.4697
KRT16	6	0.0018276	92	-1.8523
KRTAP22-1	6	0.001882	93	-3.9413
BTG1	6	0.0019064	94	-1.0452
C9orf170	6	0.0020826	95	-4.3872
GDF3	6	0.0021828	96	-4.6869
PSMC3	6	0.0023332	97	-5.3263
GPR12	6	0.0025838	98	-1.0452
SYT2	6	0.0027342	99	-5.2158
KIAA0226	6	0.0027843	100	-1.0452

Table S1. Top 100 positively ranked genes enriched in Shiga toxin resistant HeLa cells analyzed by Model-based Analysis of Genome-wide CRISPR-Cas9 Knockout (MAGeCK). NGS sequence files (Control and Resistant) were demultiplexed according to their 8-base barcodes. Non-targeting controls were used for normalization and sequence alignment was done by bowtie2.

Name	Sequence	Product Name
5’AHRF	5’-GGATGGTGATGTGCCTAATAATATAAC	5’PCR
5’AHRR	5’-TTAGCCCATACTCTTTAGTTTGGATG	
MAHRF	5’-GTACCAAATTGAACTCAAGGAGAAA	Middle PCR
MAHRR	5’-ACCATTTGATTTAGGGTAACTTCCA	
3’AHRF	5’-ACCATTAAGCTTAGAGAAGTCAAAGAG	3’PCR
3’AHRR	5’-ACTAAGACACAAACCCACACATTAG	
5’SPTLC1F	5’- TAATATCACCGTCTACTGGTAGG	5’PCR
5’SPTLC1R	5’- CCTGCATATACTGCCTCCTGTGA	
MSPTLC1F	5’- ATAAATGAATTGTATTTAGGGGG	Middle PCR
MSPTLC1R	5’- CTAATCATTATTACCTGCTGAGG	
3’SPTLC1F	5’- CTGTAATGTAAGGAAATCAAGGG	3’PCR
3’SPTLC1R	5’- CTAATCATTATTACCTGCTGAGG	

Table S2. List of PCR primers used to generate PCR products in Figure S6 and S7.

	Assay IDs	Gene Symbol
1	Hs00272311_m1	SPTLC1
2	Hs01027014_m1	SPTLC2
3	Hs00370543_m1	SPTSSA
4	Hs01048929_m1	KDSR
5	Hs00371958_g1	CERS2
6	Hs00916612_m1	UGCG
7	Hs00941041_m1	B4GALT5
8	Hs00213726_m1	A4GALT
9	Hs01060665_g1	ACTB

Table S3. List of probes for each target genes used in the qRT-PCR assays in HeLa cells.

	Assay IDs	Gene Symbol
1	Mm00447343_m1	Sptlc1
2	Mm00448871_m1	Sptlc2
3	Mm01267361_g1	Sptssa
4	Mm01290268_m1	Kdsr
5	Mm03024093_mH	Cers1
6	Mm01258345_g1	Cers2
7	Mm03990709_m1	Cers3
8	Mm00482658_m1	Cers4
9	Mm00510998_m1	Cers5
10	Mm00556165_m1	Cers6
11	Mm00492146_m1	Degs1
12	Mm00495925_m1	Ugcg
13	Mm01307145_m1	A4galt
14	Mm00480147_m1	B4galt5
15	Mm02619580_g1	Actb

Table S4. List of probes for each target genes used in the array card.

Supporting Experimental Procedures

Mammalian cell growth and conditions

HeLa cells stably expressing Cas9 endonuclease were purchased from GeneCopoeia (Cat# SCL-03-CA3). Cells were grown based on the manufacturer's protocol. All cells transfected with plasmids carrying sgRNAs were maintained in Dulbecco's Modified Eagle Medium (Life Technologies cat# 10569-044) selection media containing 200 µg/ml G418 (ThermoFisher Scientific, cat# 10131027).

Generation of KO cells

HeLa cells stably expressing Cas9 were transfected (using Lipofectamine 3000 from ThermoFisher Scientific, cat# L3000008) with either *SPTLC1* CRISPR sgRNA (5'-AAAGAAGTCTGATTATCCAG-3') or *AHR* CRISPR sgRNA (5'-GTTGTCACTACAGATGCTT-3') in pGS-gRNA-Neo vector purchased from GenScript. Stable and functional *SPTLC1* or *AHR* KO clones were selected after treating the transfected cells with media containing 400 µg/ml G418 for 2 weeks. Single clones were isolated using a cloning cylinder. A single clone from each KO pool was selected based on a complete lack of the respective protein expression by immunoblot. Control cells were generated by transfecting a non-targeting control sgRNA (5'- ACGGAGGCTAAGCGTCGCAA) plasmid (cat# U6626CC300-22) in HeLa cells stably expressing Cas9, followed by selection of a single clone. KO cells were validated by Western blotting. PCR was performed to ensure that there were no gross alterations introduced at the targeted loci (Fig. S5 and S6) (PCR primers are listed in Table S2).

Cell viability assay

Cell viability assays were performed using the PrestoBlue Cell Viability Reagent for Microplate Protocol (ThermoFisher, Cat# A13261). Cells (0.1×10^6) were seeded into a 24-well plate and kept overnight under optimal growth conditions for HeLa cells prior to assaying.

Flow cytometry

Cells (5×10^5) were stained with unconjugated anti-CD77 antibody (BioLegend, Cat# 357102), washed in 2% BSA/PBS, stained with a secondary antibody conjugated to phycoerythrin (Life Technologies, Cat# M31504), and then washed again in 2% BSA/PBS. The cells were analyzed on the BD FACSAria I flow cytometer (BD Biosciences). Cells with a fluorescence of more than two standard deviations (SD) above unstained control cells were defined as positive.

Immunoblot analysis

Cells were lysed using RIPA buffer (Thermo Fisher, Cat# 89901) using manufacturer's protocol. Halt Protease Inhibitor Cocktail was added to the cell lysis buffer (Thermo Fisher, Cat# 78429). Mouse tissues were homogenized, using a handheld homogenizer in RIPA buffer by the same procedure described above. Proteins were resolved in a 4–12% Bis-Tris NuPAGE gel (ThermoFisher Scientific) SPTLC1 and β-actin were detected by sc-374143 and sc-37413 primary antibodies from Santa Cruz Biotechnology. Primary antibody to both human and mouse AHR was purchased from Biolegend (cat# 694502). HRP conjugated secondary antibodies were used for detection.

Lipid analysis

Lipid extracts of HeLa cells (5×10^6 per sample) grown on selection media or mouse tissue homogenates (1 mg) were analyzed by HPLC-tandem MS in the Lipidomics Core at the Medical University of South Carolina on a ThermoFisher Scientific TSQ Quantum Access Max Triple Quadrupole Mass Spectrometer with the ThermoFisher Scientific Vanquish UHPLC Chromatography System. Lipid values from HeLa cells were normalized to cellular phosphate, mouse tissues were normalized to per milligram of protein. Lipid values from plasma samples were normalized to volume (100 µl).

RT-qPCR

Total RNA from HeLa cells and from mouse tissues was extracted using the miRNeasy Mini Kit (QIAGEN, Cat# 1038703). cDNAs were synthesized from 4 µg of total RNA using SuperScript IV Vilo Master Mix (Invitrogen, Cat# 11766050). Each RT-qPCR assay was done with 100 ng of cDNA from HeLa cells and detected by QuantStudio 3 from ThermoFisher (Table S3).

In mouse, RT-qPCR was done using Custom TaqMan Array Cards (ThermoFisher, cat# 4346799). cDNA (150 ng) from each biological replicate from each group was used for detection of genes in the sphingolipid biosynthetic pathway (Table S4) using the QuantStudio 7 (ThermoFisher).

LightSwitch promoter assay

Reporter constructs were prepared using the LightSwitch Luciferase Vector GoClone promoters for *SPTSSA*, *CYP1A1*, and *ACTB* (Active Motif). Control and *AHR* KO HeLa cells were transfected following the LightSwitch Luciferase Assay for GoClone Reporter Constructs manufacturer's protocol (Active Motif, cat# S714555 (*CYP1A1*), cat# S708655 (*SPTSSA*), Cat# S717678 (*ACTB*)). Luciferase assays were conducted following the LightSwitch Luciferase Assay Reagent protocol (Active Motif, Cat# LS010), with activities normalized to *ACTB* promoter activity.

ChIP-qPCR

ChIP was performed using the ChIP-IT Express Enzymatic Kit (Active Motif, cat# 53009) following the manufacturer's protocol, using an equal amount of sheared chromatin and 10 µg of anti-human AHR antibody (Santa Cruz Biotechnology; Cat# SC-133088) or anti-mouse IgG2b antibody (Santa Cruz Biotechnology; Cat# SC-69817). Immunoprecipitated DNA was purified using the ChIP DNA Purification Kit (Active Motif, # 58002) following the manufacturer's protocol and quantitated with the PowerUp SYBR Green Master Mix (ThermoFisher, cat#A25741) using gene-specific primers (QIAGEN).

Mammalian cell growth and conditions

HeLa cells stably expressing Cas9 endonuclease were purchased from GeneCopoeia (Cat# SCL-03-CA3). Cells were grown based on the manufacturer's protocol. All cells transfected with plasmids carrying sgRNAs were maintained in Dulbecco's Modified Eagle Medium (Life Technologies cat# 10569-044) selection media containing 200 µg/ml G418 (ThermoFisher Scientific, cat# 10131027).

Generation of KO cells

HeLa cells stably expressing Cas9 were transfected (using Lipofectamine 3000 from ThermoFisher Scientific, cat# L3000008) with either *SPTLC1* CRISPR sgRNA (5'-AAAGAAGTCTGATTATCCAG-3') or *AHR* CRISPR sgRNA (5'-GTTGTCACTACAGATGCTT-3') in pGS-gRNA-Neo vector purchased from GenScript. Stable and functional *SPTLC1* or *AHR* KO clones were selected after treating the transfected cells with media containing 400 µg/ml G418 for 2 weeks. Single clones were isolated using a cloning cylinder. A single clone from each KO pool was selected based on a complete lack of the respective protein expression by immunoblot. Control cells were generated by transfecting a non-targeting control sgRNA (5'- ACGGAGGCTAAGCGTCGCAA) plasmid (cat# U6626CC300-22) in HeLa cells stably expressing Cas9, followed by selection of a single clone. KO cells were validated by Western blotting. PCR was performed to ensure that there were no gross alterations introduced at the targeted loci (Fig. S5 and S6) (PCR primers are listed in Table S2).

Cell viability assay

Cell viability assays were performed using the PrestoBlue Cell Viability Reagent for Microplate Protocol (ThermoFisher, Cat# A13261). Cells ($0.1x10^6$) were seeded into a 24-well plate and kept overnight under optimal growth conditions for HeLa cells prior to assaying.

Flow cytometry

Cells ($5x10^5$) were stained with unconjugated anti-CD77 antibody (BioLegend, Cat# 357102), washed in 2% BSA/PBS, stained with a secondary antibody conjugated to phycoerythrin (Life Technologies, Cat# M31504), and then washed again in 2% BSA/PBS. The cells were analyzed on the BD FACSAria I flow cytometer (BD Biosciences). Cells with a fluorescence of more than two standard deviations (SD) above unstained control cells were defined as positive.

Immunoblot analysis

Cells were lysed using RIPA buffer (Thermo Fisher, Cat# 89901) using manufacturer's protocol. Halt Protease Inhibitor Cocktail was added to the cell lysis buffer (Thermo Fisher, Cat# 78429). Mouse tissues were homogenized, using a handheld homogenizer in RIPA buffer by the same procedure described above. Proteins were resolved in a 4–12% Bis-Tris NuPAGE gel (ThermoFisher Scientific) SPTLC1 and β-actin were detected by sc-374143 and sc-37413 primary antibodies from Santa Cruz Biotechnology. Primary antibody to both human and mouse AHR was purchased from Biolegend (cat# 694502). HRP conjugated secondary antibodies were used for detection.

Lipid analysis

Lipid extracts of HeLa cells ($5x10^6$ per sample) grown on selection media or mouse tissue homogenates (1 mg) were analyzed by HPLC-tandem MS in the Lipidomics Core at the Medical University of South Carolina on a ThermoFisher Scientific TSQ Quantum Access Max Triple Quadrupole Mass Spectrometer with the ThermoFisher Scientific Vanquish UHPLC Chromatography System. Lipid values from HeLa cells were normalized to cellular phosphate, mouse tissues were normalized to per milligram of protein. Lipid values from plasma samples were normalized to volume (100 µl).

RT-qPCR

Total RNA from HeLa cells and from mouse tissues was extracted using the miRNeasy Mini Kit (QIAGEN, Cat# 1038703). cDNAs were synthesized from 4 µg of total RNA using SuperScript IV Vilo Master Mix (Invitrogen, Cat# 11766050). Each RT-qPCR assay was done with 100 ng of cDNA from HeLa cells and detected by QuantStudio 3 from ThermoFisher (Table S3).

In mouse, RT-qPCR was done using Custom TaqMan Array Cards (ThermoFisher, cat# 4346799). cDNA (150 ng) from each biological replicate from each group was used for detection of genes in the sphingolipid biosynthetic pathway (Table S4) using the QuantStudio 7 (ThermoFisher).

LightSwitch promoter assay

Reporter constructs were prepared using the LightSwitch Luciferase Vector GoClone promoters for *SPTSSA*, *CYP1A1*, and *ACTB* (Active Motif). Control and *AHR* KO HeLa cells were transfected following the LightSwitch Luciferase Assay for GoClone Reporter Constructs manufacturer's protocol (Active Motif, cat# S714555 (*CYP1A1*), cat# S708655 (*SPTSSA*), Cat# S717678 (*ACTB*)). Luciferase assays were conducted following the LightSwitch Luciferase Assay Reagent protocol (Active Motif, Cat# LS010), with activities normalized to *ACTB* promoter activity.

ChIP-qPCR

ChIP was performed using the ChIP-IT Express Enzymatic Kit (Active Motif, cat# 53009) following the manufacturer's protocol, using an equal amount of sheared chromatin and 10 µg of anti-human AHR antibody (Santa Cruz Biotechnology; Cat# SC-133088) or anti-mouse IgG2b antibody (Santa Cruz Biotechnology; Cat# SC-69817). Immunoprecipitated DNA was purified using the ChIP DNA Purification Kit (Active Motif, # 58002) following the manufacturer's protocol and quantitated with the PowerUp SYBR Green Master Mix (ThermoFisher, cat#A25741) using gene-specific primers (QIAGEN).
